# Leveraging Shannon Entropy to Validate the Transition between ICD-10 and ICD-11

**DOI:** 10.3390/e20100769

**Published:** 2018-10-08

**Authors:** Donghua Chen, Runtong Zhang, Xiaomin Zhu

**Affiliations:** 1 School of Economics and Management, Beijing Jiaotong University, Beijing 100044, China; 2 School of Mechanical, Electronic and Control Engineering, Beijing Jiaotong University, Beijing 100044, China

**Keywords:** ICD-11, ICD-10, Shannon entropy, validation, transition

## Abstract

This study aimed to propose a mapping framework with entropy-based metrics for validating the effectiveness of the transition between International Classification of Diseases 10th revision (ICD-10)-coded datasets and a new context of ICD-11. Firstly, we used tabular lists and mapping tables of ICD-11 to establish the framework. Then, we leveraged Shannon entropy to propose validation methods to evaluate information changes during the transition from the perspectives of single-code, single-disease, and multiple-disease datasets. Novel metrics, namely, standardizing rate (SR), uncertainty rate (UR), and information gain (IG), were proposed for the validation. Finally, validation results from an ICD-10-coded dataset with 377,589 records indicated that the proposed metrics reduced the complexity of transition evaluation. The results with the SR in the transition indicated that approximately 60% of the ICD-10 codes in the dataset were unable to map the codes to standard ICD-10 codes released by WHO. The validation results with the UR provided 86.21% of the precise mapping. Validation results of the IG in the dataset, before and after the transition, indicated that approximately 57% of the records tended to increase uncertainty when mapped from ICD-10 to ICD-11. The new features of ICD-11 involved in the transition can promote a reliable and effective mapping between two coding systems.

## 1. Introduction

The International Classification of Diseases (ICD) is a global standard for diagnostic health information [1]. The ICD developed by the World Health Organization (WHO) enables sustainable and systematic recording, analysis, interpretation, and comparison of mortality and morbidity rates of different countries at various time points. Over the past 20 years, the 10th revision of the ICD (ICD-10) has been widely utilized in classifying healthcare information. The 11th revision of the ICD (ICD-11) was formally released on 18 June 2018 for testing and implementation, in accordance with specific timelines and requirements of different countries. The development of the new ICD standards will revolutionize the global medical informatics with opportunities and challenges for quality and safety in the next several decades [2].

The ICD standards have been used in medicine and healthcare for over a century. The first ICD standards initially focused on the statistics of causes of death. In 1946, the Interim Commission of the WHO took over the revision of the ICD and introduced a method for disease classification. At present, the most widely used version of the ICD is ICD-10, which was proposed in 1989. In contrast to ICD-10, ICD-11 is established upon ontology models. Some of the value sets in ICD-11 are derived from external ontologies, such as the Systematized Nomenclature of Medicine—Clinical Terms (SNOMED CT) [3]. The WHO released ICD-11 in 2018 to provide an international standard for disease classification in the 21st century [4]. Subsequently, ICD-11 will be submitted to the 144th Executive Board Meeting in January 2019, and then to the 72nd World Health Assembly in May 2019. The member states of the WHO will begin reporting the use of ICD-11 on 1 January 2022, after the endorsement.

The structure and design of the newly proposed ICD-11 are based on clinical practices over the past few decades and differ considerably from those of the previous versions of the ICD [5,6]. For example, ICD-11 provides novel concepts that allow multiple purposes for disease classification. Moreover, the code scheme that contains stem and extension codes in the ICD-11 is also new to users of ICD-10. Many challenges in the transition from ICD-10 to ICD-11 exist, such as previous practices of building a bridge between the ICD-9 content model (CM) and ICD-10-CM, due to the complexity of the structure and guidelines in ontology-based ICD-11 [7]. In addition, the lack of proper metrics on validating information changes in a different context of ICD standards also prevents us from utilizing new features of ICD-11 from the perspective of using massive ICD-10-coded data [8,9]. Therefore, the utilization of existing ICD-10-coded data in adapting new features of ICD-11 for global service needs in healthcare is essential [10].

The transition from ICD-10 to the new features of ICD-11 complicates further development of support tools for medical information systems. The foundation component (FC) [11] and the CM [12] are key components according to the reference guidance of the ICD-11. The FC is a multidimensional collection of all ICD entities in ICD-11. The entities cover diseases, disorders, injuries, external causes, signs, and symptoms. Some entities may be broad (for example, “injury of the arm”), whereas others may be highly detailed (such as “laceration of the skin of the thumb”). The CM defined by 13 attributes provides background knowledge of each ICD entity to allow for computerization. Generally, ICD-10 is organized in a tree-type structure with stems and branches. The structure details disease classification in healthcare layer by layer, until the layer reaches a unique disease code. However, the ICD-11 can be utilized for multiple parenting of diseases. For example, type-2 diabetes mellitus in ICD-11 belongs to different types of endocrine diseases and startup mortality list. A formal concept analysis in ICD-11 will greatly influence various types of medical and health data in the future [13]. In summary, reducing the gap during the transition from ICD-10 to ICD-11 is important to utilize existing ICD-10-coded data [14,15].

The rest of this paper proposes an ICD mapping framework, which facilitates transition between ICD-10-coded datasets and ICD-11-coded datasets in Section 2. Three metrics based on the Shannon entropy theory [16] to evaluate information changes during the transition are proposed. Then, we utilize an ICD-10-coded dataset to examine the performance of the method in terms of transforming ICD-10 codes to ICD-11 codes, through the proposed metrics in the process of adapting new features of ICD-11. Finally, we discuss and conclude the work. The purpose of this study is to help researchers to perform data-analysis with medical data from different ICD systems, which is very useful in enhancing clinical decision support based on existing ICD-coded data in the new standards.

## 2. Materials and Methods

### 2.1. ICD Mapping Framework

**Figure 1:**
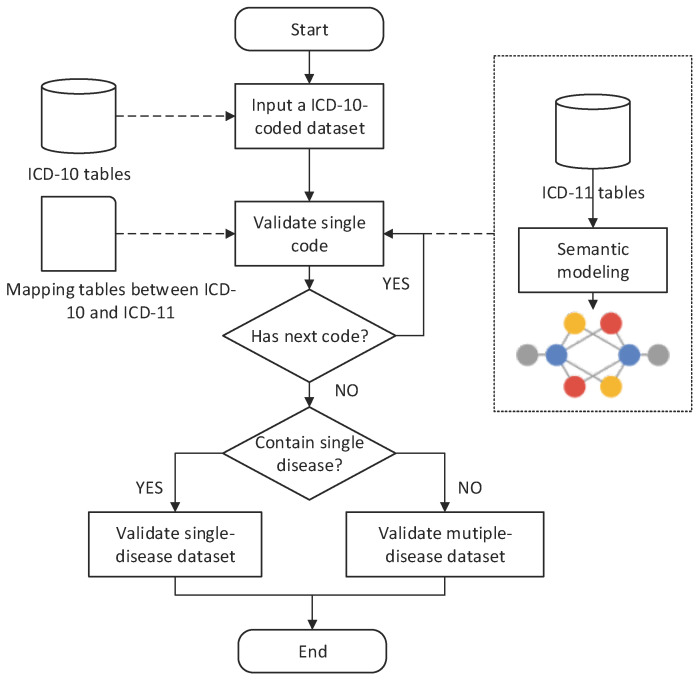
Overview of an International Classification of Diseases (ICD) mapping framework.

A framework that can ensure the effectiveness of the transition between different coding systems is essential [17]. For example, mapped ICD codes are also reused to ensure high-quality analysis, such as the investigation of the causes of death [18]. Our ICD mapping framework was established on ICD-related tables from ICD-10 and ICD-11, as shown in Figure 1. The ICD-10 tables include chapter, section, and code tables, whereas those of ICD-11 include simple tabulation and corresponding ICD-10-to-ICD-11 mapping tables. In addition, some ICD-10-coded datasets from our cooperative hospitals were collected for testing. The detailed roles of the datasets in the framework are as follows.

First, the ICD-11 released from the WHO in 2018 provides a simple tabulation with an easy-to-access structure to obtain relevant information of parents and children of specific ICD codes. Therefore, we construct a database to support rapid query of relevant information for the framework. In addition, a mapping table between ICD-10 and ICD-11, also acquired from the release of ICD-11, can be used to obtain one-to-one and one-to-multiple mapping relationships from ICD-10 to ICD-11 codes.

Second, the datasets of ICD-10 provide the basis of initial validation on ICD-10-coded datasets. The ICD-10-coded datasets were initially evaluated using the original ICD-10 tables to provide a benchmark for future validation, such as validating 30-day mortality across ICD-9 and ICD-10 [19]. Moreover, the tables in the ICD-10 provide comparison between standardized terms and the terms in the datasets in the context of ICD-10.

Lastly, ICD-10-coded datasets were prepared to validate the transition during mapping. Each ICD-10-coded dataset contained at least two columns, namely, textual diagnosis information and the corresponding ICD-10 code. The information in the dataset varied depending on the digital environments and use of different versions in various countries.

In summary, the aforementioned datasets from ICD-10 and ICD-11 provided the basis of the framework used in the validation between codes. A modification of the datasets was necessary before transition. The framework mapped ICD-10 codes to ICD-11 codes sequentially from code, single-disease dataset, and multiple-disease dataset levels.

### 2.2. ICD Validation Methods

The validation of ICD-10-code datasets during ICD mapping aims to examine legitimacy of the dataset for secondary use in ICD-11. Based on the aforementioned framework, we introduced the Shannon entropy into the process of validating information changes during the transition.

#### 2.2.1. Single-Code Validation (SV)

The SV during mapping between ICD-10 and ICD-11 verified the changes of information between ICD codes. Three entropy-based metrics applied in the SV were the standardizing rate (*SR*), uncertainty rate (*UR*), and information gain (*IG*) among codes. Developing such metrics and automated tools is useful for future use of consistent discovery of disease and financial analyses [20].

Validation with the *SR* refers to the examination of various ICD-10-coded datasets from different digital environments, to evaluate the difference of codes in transforming the datasets into standardized ones [21]. Generally, ICD-10-coded datasets vary from different hospitals and countries due to the consideration of quality and safety [22]. In code standardization, the mapping tables that preserve the mapping relationship, such as *c*_10_ → {*c*_11_(*i*) | 0 ≤ *i* ≤ *N*_11_}, where *N*_11_ is the number of optional ICD-11 codes in the tables, are used. On the basis of the tables and a specific ICD-10 code, a corresponding ICD-10 code with the longest length of code in the mapping table provided by WHO is determined as a standardized ICD-10 code. Assume that an ICD-10-code in the dataset is *c*_10_*^t^*, and an ICD-10 code in the mapping table is *c*_10_. Then, an *SR* based on the longest common subsequence (*LCS*) [23], which examines the information changes in the dataset during the process using distance metrics is obtained as follows:

(1) $$SR=1-\frac{\left|LCS\left(c_{10},c_{11}\right)\right|}{\max(n,m)},$$

where the *LCS* distance between *c*_10_ and *c*_11_ is *n* + *m* − 2 |*LCS*(*c*_10_, *c*_11_)|, *n* is the number of the bits in an ICD-10 code, and *m* is the number of bits in a mapped ICD-10 code. If *SR* = 0, then the two codes are similar; therefore, code standardization is unnecessary. Otherwise, a large *SR* means a considerable difference between two codes. Thus, the *SR* in Equation (1) validates the examination of the information changes in code standardization. 

Validation with the *UR* refers to the utilization of the Shannon entropy to examine the degree of uncertainty of mapping results from standardized ICD-10 to ICD-11. Generally, improper mapping often leads to the corruption of dataset validity. An ICD-10 code is associated with multiple ICD-11 codes, with their corresponding definitions. For example, an ICD-10 code “I25.1” in the mapping table is associated with two ICD-11 codes “BA80” and “BA8Z.” The example prevents seamless transition from ICD-10-coded datasets to the ICD-11-based context. To evaluate the degree of uncertainty in such cases, we used *UR* in Equation (2) to illustrate the entropy changes in the process. Assume that an ICD-10 code *c*_10_ is ready to map a possible set of ICD-11 codes {*c*_11_(*i*) | 1 ≤ *i* ≤ *M*}, where *M* is the number of ICD-11 code candidates, and a set of probabilities of the ICD-11 codes is {*p_i_* | 1 ≤ *i* ≤ *M*}. According to the Shannon entropy, the *UR* of the *c*_10_ → *c*_11_ process between codes can be expressed as follows:

(2) URc10→c11=∑1MpiI(c→10c11i)=∑1Mpilog1pi,

where *I*(*c*_10_ → *c*_11_) is the self-information of the *c*_10_ → *c*_11_ process, and the default log algorithm is log_2_. If *M* = 1, then *UR* = 0; thus, the ICD-10 code is precisely mapped to the correct ICD-11 code. The probability *p_i_* in Equation (2) varies from the context of ICD-11. If all probabilities of ICD-11 codes are equal, then the *UR* in Equation (2) reaches a maximum entropy log_2_*N*, where ∀*p* = *1*/*N*.

The maximum *UR* in Equation (2) indicates that the mapping result reaches the greatest uncertainty [24], which refers to the most probable inaccurate transition on ICD-10-coded datasets, because the framework cannot determine an optimal choice based on equivalent probabilities. Therefore, some rules in differentiating each *p_i_* are necessary. The *FC* in ICD-11 provides the rules by transforming the guidelines of ICD-11 coding into logical rules used in a coding system [25].

To increase the sensitivity of *UR* in evaluating uncertainty, we considered three cases, namely, Cases 1–3. In Case 1, the *p_i_* in Equation (2) is assigned an average probability as in 1/*M*. In Case 2, considering the text similarity of titles of ICD codes based on normalized Levenshtein metrics [26] provides additional information in determining the *p^i^* in Equation (2). In Case 3, the *p^i^* in Equation (2) is assigned randomly, assuming we do not consider the context factors of ICD-11. We denote the determination of probabilities in the three cases as a function *prob*(). Therefore, we have a multiple-code selection algorithm, as shown in Algorithm 1.


**Algorithm 1. Multiple-code selection algorithm**
**Input:** an ICD-10 entity *c*_10_, a list of ICD-11 entities {*c*_11_(*i*)|1≤*i*≤*M*} **Output:** entropy of the process *UR*, optimal ICD-11 code *c*_11_* Let
*pro* ← {*p_i_*|1≤*i*≤*M*} and *M* ← the number of ICD-11 code candidates **for each**
*c*_11_(*i*) **in** {*c*_11_} **do** $p_{i}\overset{prob()}{\leftarrow }\{\begin{array}{c} 1/M,\\ NormalizedLevenshtein(c_{10},c_{11}\left(i\right)),\\ Random(0,1), \end{array}\begin{array}{c} \mathrm{In}\;Case^{}1\\ \mathrm{In}\;Case^{}2\\ \mathrm{In}\;Case^{}3 \end{array}$ **end for**
**for each**
*c*_11_
**in** {*c*_11_} **do** $p_{i}\leftarrow \frac{p_{i}}{\sum_{1}^{M}p_{i}}$ **end for** **return**
$UR=\sum_{i=1}^{M}p_{i}\log_{2}\frac{1}{p_{i}}$, $c_{11}{}^{*}=c_{11}\left[i{]}^{}\;{\mathrm{where}}^{}\;s.t.\max(p_{i}\right)$


**Figure 2:**
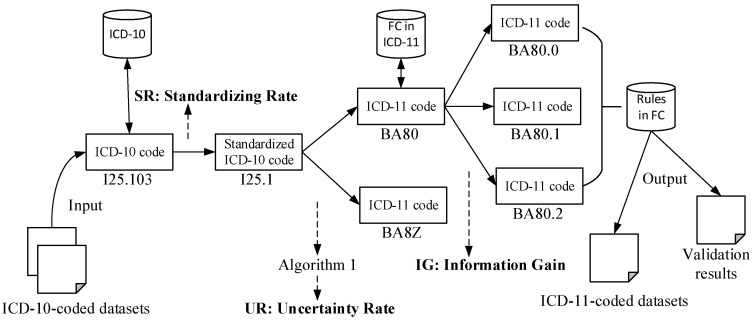
Flowchart of the Single-Code Validation (SV) in a mapping framework from ICD-10 to ICD-11.

Finally, after a proper ICD-11 code *c*_11_^*^ to represent the original ICD-10 code *c*_10_ is determined using Algorithm 1, we have *IG*(*c*_10_, *c*_11_^*^) from code level, as follows:

(3) $$IG(c_{10}|c_{11}{}^{*})=I\left(c_{10}\right)-\sum_{v\in Values\left(A\right)}\frac{\left|S_{v}\right|}{\left|S\right|}I\left(c_{11}{}^{*}\right),$$

where *I*(*c*_10_) = *UR* is an entropy of an ICD-10 code to *c*_11_^*^, *I*(*c*_11_^*^) is an entropy of the selected ICD-11 code *c*_11_^*^ in the context of ICD-11, *S_v_* is the possible ICD-11 candidate in a mapping code set *A*, *S* is the total number of ICD candidates, and |*S_v_*|/|*S*| is the percentage of selected codes in the set. Thus, we have *IG*(*c*_10_, *c*_11_) bits of information regarding the code-level transition. Different *IG*s, such as *IG*_1_, *IG*_2_, and *IG*_3_, for Cases 1–3, are obtained respectively. On the basis of Equations (1) to (3), a flowchart of the SV is developed, as shown in Figure 2. 

In summary, we proposed the SV to validate the transition between ICD-10 and ICD-11 from code level. The *SR*, *UR*, and *IG* metrics were proposed to evaluate the changes of entropy during the code-to-code process. 

#### 2.2.2. Single-Disease Dataset Validation (SDV)

Based on the SV with three metrics, we proposed SDV to validate the records in a dataset focused on a single type of disease or a group of similar diseases. For example, a single-disease dataset includes patients who suffer from liver disorders. The goal of validating single-disease datasets is to examine whether the mapped ICD-10 codes were in a subset of ICD-11 disease-based domains. One of the methods of validating the dataset with ICD codes was through arithmetic averaging of the *UR*s in the SV. Assume that *H_c_* represents a *UR* obtained in the SV. Then,

(4) $$H_{sd}=\frac{\sum_{1}^{N}H_{c}}{N},$$

where *N* is the number of records in a single-disease dataset *sd*. However, the methods of evaluating the *sd* have drawbacks because the average entropy of ICD codes in a dataset cannot represent the entire distribution of validation results within a dataset. Another method to analyze *sd* is to evaluate the sample distribution of the entropy calculated in Equation (1). Suppose that *H_sd_* is a random variable *x*. We use the cumulative distribution function (CDF), that is, *F_x_*(*x*) = *P*(*X* ≤ *x*), to describe the probability distribution of the dataset. The CDF that illustrates percentage of samples provides an intuitive charting tool to validate the mapping results in a single-disease dataset. 

We can also use a single metric to validate the effectiveness of the mapping process. By selecting a confidence interval *e* of *UR*, the distribution of entropy within a single-disease dataset can be depicted. Assume that an entropy is in a range of *e* = [*e*_min_, *e*_max_], where *e_min_* and *e_max_* represent the minimum and maximum values of entropy, respectively. If a mapping result falls in *e*, then we represent the mapping accuracy for the dataset as follows:

(5) $$accuracy=\frac{n_{c}}{n_{c}+n_{e}},$$

where *n_c_* is the number of the properly mapped record, and *n_e_* is the number of incorrectly mapped records. 

#### 2.2.3. Multiple-Disease Dataset Validation (MDV)

An ICD-10-coded dataset with multiple diseases may have an influence on validation results. For example, ICD codes of chapters have different influences in the entropy during mapping. On the basis of the SDV, we proposed cluster-based MDV. Thus, we have the entropy of cluster *i* in a multiple-disease dataset as follows:

(6) $$H\left(c_{i}\right)=-\sum_{j=1}^{\left|diseases\right|}p_{i,j}\log\left(p_{i,j}\right),$$

where *p_i_*_,*j*_ = *m_i_*_,*j*_/*m_i_* is a probability that a record from cluster *i* belongs to class *j*, in which *m_i_*_,*j*_ is the number of instances in cluster *i* with class, and *m_i_* is the number of records in cluster *i*. Then, the average entropy of a clustering is obtained as follows:

(7) $$\bar{H\left(c\right)}=\sum_{1}^{K}\frac{m_{i}}{m}H\left(c_{i}\right),$$

where *m_i_* is the number of ICD codes in cluster *i*, and *m* is the number of all records in the dataset. In the MDV, the IG of the transition *IG*(*d*,{*d_s_*}) can be expressed as

(8) $$IG\left(d,\left\{c\right\}\right)=H\left(d\right)-\bar{H\left(c\right)},$$

where *H*(*d*) is the entropy of the multiple-disease dataset using the SDV. 

In summary, on the basis of the SV and the SDV, the MDV allows the mapping framework to validate massive records of datasets with multiple diseases by considering the difference of multiple diseases in the context of ICD-11. 

## 3. Results

### 3.1. SR during Standardization

**Figure 3:**
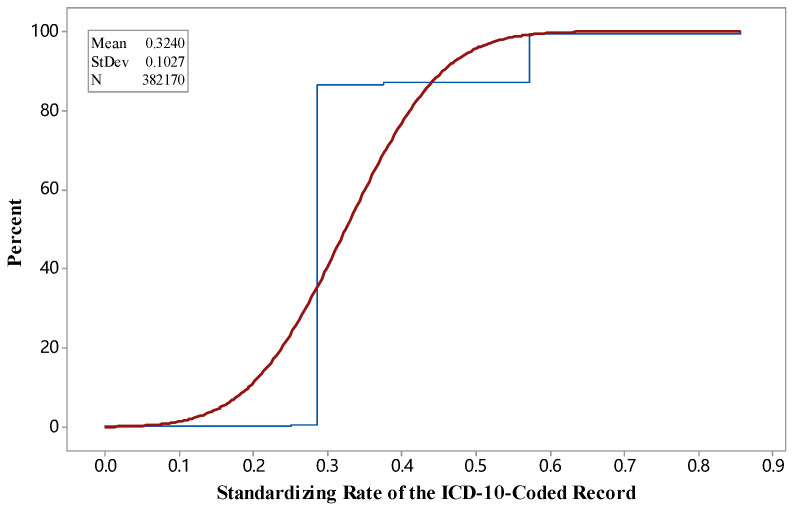
Empirical cumulative distribution of frequency of standardizing rate (*SR)* in an ICD-10-coded dataset.

We examined the *SR* on evaluating information changes when transforming the codes into standardized ones by analyzing 377,589 coded records in our test dataset. Figure 3 illustrates an empirical CDF of the *SR* in the dataset. 

Generally, large *SR*s refer to a considerable difference between an original code and a standardized one. Thus, when *SR* = 0, no difference between the two codes is observed. As shown in Figure 3, most of *SR*s fall into the range of [0.0, 0.6]. The result of *SR*s demonstrated that in ICD mapping, a considerable difference (of 60% of records) between the two codes existed, thereby affecting the efficiency of ICD mapping. 

### 3.2. UR during Validation 

**Figure 4:**
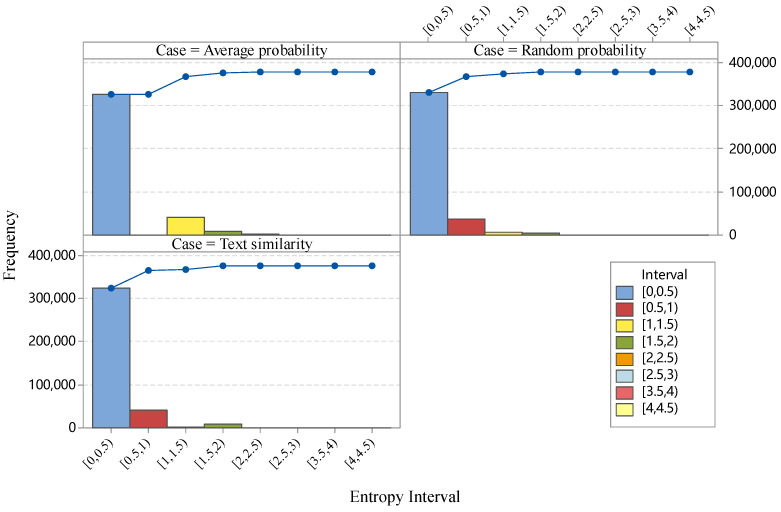
Pareto chart of the entropy interval of the three cases in SV.

On the basis of the standardized results in Figure 3, we implement the SV in the validation of the test dataset. We analyzed the experimental results of the *UR* to examine the aforementioned three cases in the mapping process. Figure 4 presents the Pareto chart of the entropy interval of Cases 1–3 in the SV. 

The entropy-based *UR* in the SV provides a metric to examine the degree of uncertainty during the mapping process between two coding systems. Large *UR*s indicate considerable uncertainty of transition. As shown in Figure 4, three cases are considered. The difference of the cases in probability calculation of selecting ICD-11 codes was analyzed. As shown in Figure 4, *UR*s with 86.21% ICD-coded records of the dataset in all three cases are in the range of [0.0, 0.5], which indicates that the dataset achieves high *UR*s and can be mapped to ICD-11 codes precisely. However, 17.79% of the ICD-10-codes cannot be mapped to ICD-11 codes precisely. The comparison of the Pareto charts of the three cases shows that the *UR* in [0.0, 0.5] is constant, whereas the distribution of *UR*s in [0.5, +∞] varies. Therefore, the ICD-codes with high *UR*s should be examined further for future use in an automated ICD mapping process. 

### 3.3. IG during Mapping

Given that major changes exist during the revision of ICD-11, information changes of the mapped ICD-10 codes in the new context of ICD-11 must be validated. Based on the definitions of *IG*s in Equation (8), if an *IG* is less than zero, then the information of the mapped ICD-11 code contains more than that of the ICD-10 code. Table 1 provides the experimental results of *IG*s by analyzing the dataset. We divided the entire range of *IG*s gained in the process into 10 intervals of entropy and performed statistics on each interval. 

For example, given 86 coded records with two distinctive ICD-10 codes in the dataset mapped to ICD-11 codes, the *IG*s fell in the range of [−12, −10]. The frequencies in Table 1 show a different distribution of the ICD codes in different intervals of *IG* during mapping. Approximately 57% of the records in the dataset tended to reduce uncertainty during the mapping from ICD-10 to ICD-11, whereas the other records provided further information to an ICD-11 coding system. The *IG*s of approximately 50% of the records were in [0, 2], which demonstrated that the transition from ICD-10 to ICD-11 was likely to be in the same information amount. 

**Figure 5:**
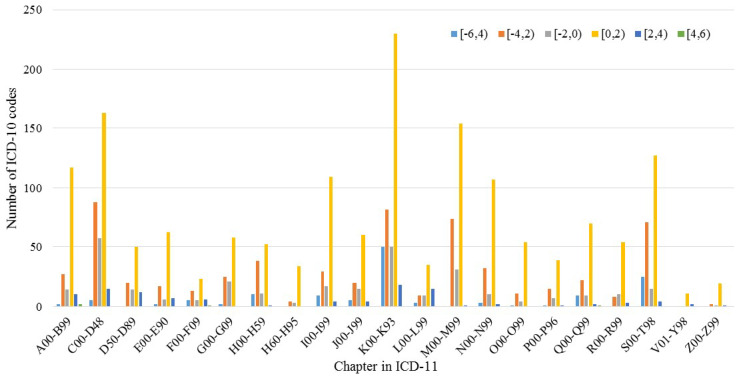
Changes of the number of ICD-10 codes in different chapters of ICD-10, over varying information-gain intervals.

Figure 5 shows an overview of the distribution of the number of ICD-10 codes in the dataset from different chapters, based on the results in Table 1. The number of ICD-10 codes changes in different chapters, over varying intervals of *IG*s. The results indicated that different multiple disease-based datasets may need to consider clustering as a first step to enable effective validation of the mapping process. 

## 4. Discussion

We proposed an ICD mapping framework and developed validation methods by leveraging existing tables from ICD-10 and ICD-11. Our method was a novel approach because we developed a mapping framework which uses existing tables of ICD-10 and ICD-11, to help utilize the existing ICD-10-coded datasets in adapting new features of ICD-11. We also proposed validation methods for evaluating information changes of codes in the mapping process during the transition, from the perspective of single-code, single-disease, and multiple-disease datasets. 

As previously stated, our method aims to provide a new way to map existing ICD-10-coded datasets to the context of newly designed ICD-11 standards. In such mapping processes, we use the Shannon theory to monitor information changes of ICD codes from single-code, single-disease, and multiple-disease levels. We obtained several findings from the experimental results. First, ICD-10 codes from the datasets for the same diseases/disorders validated using *SR* may not be in tune due to different ICD-10 implementation in different hospitals and countries. The results in Figure 3 utilized a CDF chart to illustrate such a distribution. Second, during an automated mapping process, great complexity between two coding systems was observed, such as mapping from SNOMED CT to ICD-10-CM [27] or from ICD-9 to SNOMED CT [28]. *UR* was used to quantify such information changes during the transition from ICD-10 to ICD-11 codes. Then, we illustrated three cases of determining probability based on average probability, text similarity, and random probability integrated by Algorithm 1. A huge portion of the ICD-10 codes could seamlessly be mapped to ICD-11 codes, whereas the others with high uncertainty exist in the mapping process. The small portion of the codes in Figure 3 were the key to fulfill the proper transition between ICD-coded datasets. Ideally, the transition should be seamless. However, some codes in ICD-11 context change a lot. The key to a successful transition between the datasets is to ensure proper transition between the codes with great difference. Thus, a small portion of failed transition will cause the total failure of the use of an entire medical dataset. It shows the importance of the *SR* in evaluating the similarity between codes. Verifying the ICD-10 codes with high *UR*s can be useful to have a smooth transition. When an ICD-10 code is mapped to a new ICD-11 code, the new coding scheme in the context of ICD-11 provides more details of expert knowledge than that in ICD-10, which leads to inconsistency in the medical information between two coding systems. Moreover, the validation in single- and multiple-disease levels can be different in the evaluation of *IG* between codes from different ICD standards [29]. The findings from Table 1 and Figure 5 can help instruct future applications of ICD-11. 

The release of ICD-11 in 2018 will revolutionize the development of innovation, technology, and application of medical informatics in the future [30]. Although ICD-11 is valuable to research on healthcare-related diseases, implementing the completely new ICD-11 standard in each member state of the WHO is difficult. ICD-9-CM coding alone may be insufficient to identify some specific diseases. For example, given that ICD-10 was first released more than 20 years ago, relatively more than 100 countries have reached the ICD-10 standards because the number of codes had increased from 13,000 in ICD-9 to 68,000 codes in ICD-10. In addition, doctors’ workloads had increased after the adoption of ICD-10 because patients’ diseases, diagnoses, and treatments must be recorded as accurately and precisely as possible. Meanwhile, medical institutions had to upgrade their healthcare information systems to adapt to the needs of ICD-10 coding. Substantial time and money were required to hire staff in the fields of medical research, information technology, and administration to complete the transition from ICD-9 to ICD-10. At present, ICD-11 contains approximately 269,280 codes. This number is greater than that in ICD-9 and ICD-10. It is believed that the difficulties and cost of promoting the use of ICD-11 at the beginning will also increase, but the patients and hospitals may benefit a lot through the improvement of the management level of medical informatics in the future. The revision of the existing coding systems should be automated, and redesigning the existing systems will affect the efficiency of ICD coders in the future. Therefore, utilizing and migrating existing ICD-10-coded data to a new context of ICD-11-based digital environments is likely to promote future use of historical medical data.

## 5. Conclusions

We proposed an ICD mapping framework for utilizing ICD-10-coded datasets to adapt to the new context of ICD-11. Three metrics, namely, *SR*, *UR*, and *IG*, to validate the information changes of ICD codes from the perspective of different levels in the framework were proposed. The *SR* is feasible to evaluate the information loss of ICD-10 codes, when the codes are standardized using a different method. Then, the results of calculating *UR* in the multiple-disease dataset indicated that the *UR*s of the datasets in the mapping process vary from different contexts in the ICD. We also examined the *IG* between ICD-10 codes and mapped ICD-11 codes. Our findings showed that the mapping process caused increasing uncertainty in selecting a proper candidate of ICD-11 codes related to an ICD-10 code, which should be considered when utilizing mapping and migration between two ICD coding systems in the future. 

## Figures and Tables

**Table 1:** Frequency of different ranges of information gain (IG) during mapping.

Interval of Information Gain	Frequency	Percentage (%)	Number of ICD-10 Codes
[−12, −10)	86	0.00	2
[−10, −8)	0	0.00	/
[−8, −6)	0	0.00	/
[−6, −4)	63,256	0.17	107
[−4, −2)	101,378	0.27	523
[−2, 0)	50,695	0.13	264
[0, 2)	156,884	0.42	1405
[2, 4)	5277	0.01	92
[4, 6)	12	0.00	4
[6, 8)	1	0.00	1
